# Supplementary material for: Real-Time fMRI Neurofeedback Training of Selective Attention in Older Adults
Source: Brain Sci. 2024 Sep 18;14(9):931. doi: 10.3390/brainsci14090931 (PMC11430676; doi:10.3390/brainsci14090931)
Supplement: Supplementary file 1 [file brainsci-14-00931-s001.zip › brainsci-3133114-supplementary.pdf]

## Supplementary Materials

*Comparison of Cognitive Status between Participants with PD and SCD and the Remaining Older Adult Sample*

We conducted Kruskal-Wallis H tests to compare TICS[39] and MoCA[40] scores between participants with PD, SCD, and the rest of the older adults and observed no significant differences on either the TICS ($\chi^2(2) = 1.127$, $p = 0.57$) or the MoCA ($\chi^2(2) = 1.003$, $p = 0.61$) among these three groups. Further, we compared the three groups on four cognitive measures from the NIH Cognition Toolbox[42] and found no group differences: Flanker Inhibitory Control and Attention Test, $\chi^2(2) = 4.882$, $p = 0.09$; Dimensional Change Card Sort Test, $\chi^2(2) = 2.161$, $p = 0.34$; List Sorting Working Memory Test, $\chi^2(2) = 1.425$, $p = 0.49$; and Pattern Comparison Processing Speed Test, $\chi^2(2) = 4.176$, $p = 0.12$).

*Comparison of Behavioral Performance (Reaction Time and Response Accuracy) between Participants with PD and SCD and the Remaining Older Adult Sample*

To demonstrate comparability of behavioral performance in participants with PD, SCD, and the rest of the older adults, we conducted Kruskal-Wallis tests on both reaction time and response accuracy in the MSIT across the neurofeedback training runs. Neither for reaction time ($\chi^2(2) = 2.43$, $p = 0.29$) nor for response accuracy ($\chi^2(2) = 0.09$, $p = 0.96$) were there significant differences between the three groups.

**Table S1.** Means (Standard Deviations) of Reaction Time and Response Accuracy in the MSIT across Neurofeedback Training Runs among Older Adults.

| | Older<br>(*N*= 18) | PD<br>(*N* = 5) | SCD<br>(*N* = 4) |
|---|---|---|---|
| Reaction Time | 0.99 (0.16) | 1.12 (0.23) | 0.91 (0.09) |
| Accuracy | 28.56 (9.16) | 26.39 (9.88) | 30.98 (3.76) |

*Localizer Task for Determination of dACC ROIs*

The localizer task adopted the MSIT paradigm[20] in a block design. In particular, four stimulus types were presented in a total of 24 blocks across two functional runs using a fixed order of blocks (three blocks for each stimulus type per run; Figure S1A). Within each block, there were 20 trials and each trial lasted for 1 second (during which participants gave their response), followed by a fixation cross presented for 0.5 seconds. The inter-block interval was 7.5 seconds, during which a fixation cross was presented. To determine dACC ROIs, we used two of the four stimulus types: *control trials (i.e., Type 1* stimuli; identical to the control trials used in Bush & Shin[20]) and *interference trials (i.e., Type 4* stimuli; identical to trials used during the present study's neurofeedback training). *Type 2* stimuli were the same as *Type 1* stimuli but also presented a face picture in the background[51]; *Type 3* stimuli were the same as the interference trials in Bush and Shin[20] (identical to *Type 4* stimuli without a face picture in the background).

Online processing of the localizer task was performed during MRS acquisition. We conducted a GLM analysis on the preprocessed fMRI data from the localizer task using a t-contrast (i.e., Type 4 stimuli > Type 1 stimuli) to identify brain regions with greater activity to interference than control trials. Then, for every participant we located the peak voxel within left and right dACC respectively nearby the coordinates reported in Bush et al.[8] (left dACC: MNI x = -10, y = 10, z = 49; right dACC: MNI x = 3, y = 10, z = 47) and applied a 5-mm radius sphere around these two peak voxels.

As control regions, we used a 5-mm radius sphere around the coordinates of left and right PAC based on the literature (i.e., left PAC: MNI x = -52, y = -23, z = 5; right PAC: MNI x = 55,

y = -18, z = 5)[65]. We chose PAC as control region as it is not specifically involved in selective attention[8] and/or emotion processing[50,51].

**Figure S1.** Graphical description of the localizer task. *Panel A*: The localizer task used a block design, with 3 blocks per each of the four types of stimuli presented in a fixed order. *Panel B:* Four types of stimuli based on the MSIT paradigm (see [20, 51] for details).

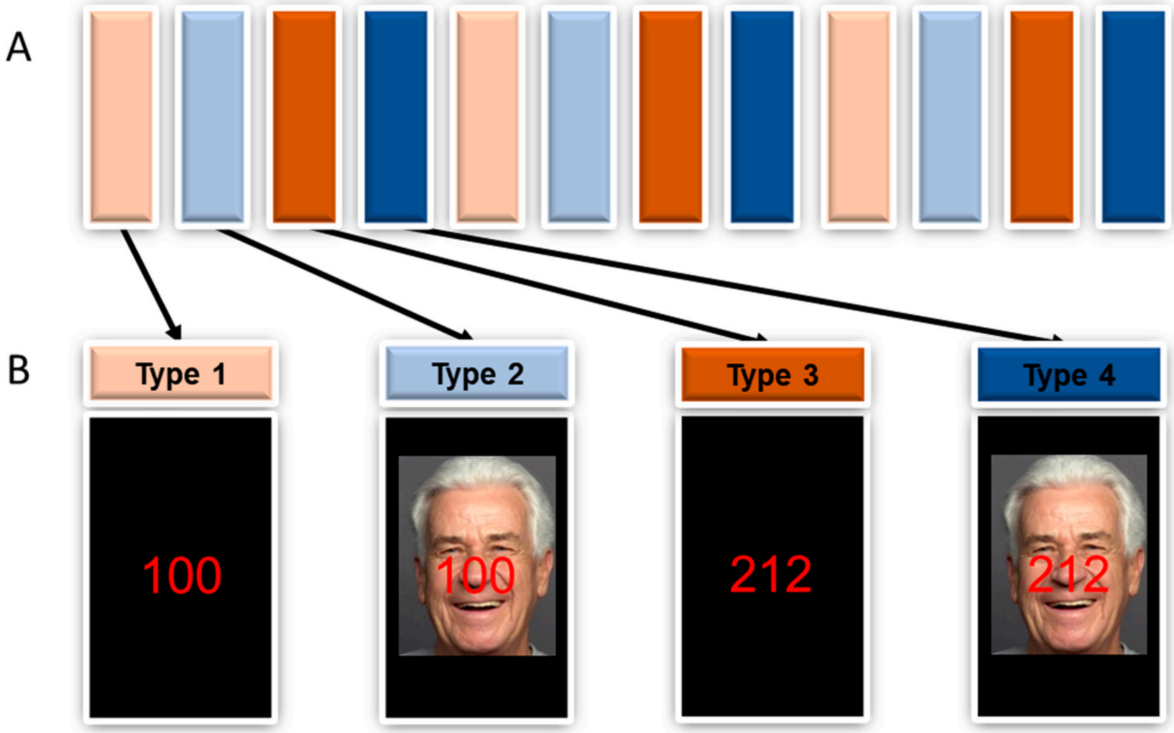

*Preprocessing of the Localizer Task and the Neurofeedback Training Data for the Offline Analysis*

The offline analysis of the localizer task and the neurofeedback training was conducted after preprocessing of the data using *fMRIPrep* 20.2.1[66] (RRID:SCR_016216; see https://fmriprep.org/en/20.2.0/citing.html for description and details), which is based on Nipype 1.5.1[67] (RRID:SCR_002502). The T1 image from the first neurofeedback training session was corrected for intensity non-uniformity (INU) with N4BiasFieldCorrection[68], distributed with ANTs 2.3.3[69] (RRID:SCR_004757). The T1 reference was then skull-stripped with a Nipype implementation of the antsBrainExtraction.sh workflow (from ANTs), using OASIS30ANTs as target template. Brain tissue segmentation of cerebrospinal fluid, white matter, and gray matter was performed on the brain-extracted T1 using FAST[70] (FSL 5.0.9, RRID:SCR_002823). A T1 reference map was computed after registration of the T1 images (after INU-correction) using mri_robust_template (FreeSurfer 6.0.1)[71]. Volume-based spatial normalization to the MNI152NLin2009cAsym standard space was performed through nonlinear registration with antsRegistration (ANTs 2.3.3), using brain-extracted versions of both the T1 reference and the T1 template images.

For images from the localizer and the neurofeedback training runs, the following preprocessing procedure was performed. First, a reference volume and its skull-stripped version were generated using a custom methodology of fMRIPrep. Susceptibility distortion correction was omitted since b0 field maps were not collected in this study. The BOLD reference was then co-registered to the T1 reference using FLIRT (FSL 5.0.9)[72] with boundary-based registration[73] cost-function. Co-registration was configured with nine degrees of freedom to account for distortions remaining in the BOLD reference. Head-motion parameters with respect

to the BOLD reference (transformation matrices, and six corresponding rotation and translation parameters) were estimated before any spatiotemporal filtering using MCFLIRT (FSL 5.0.9)[74]. BOLD runs were slice-time corrected using 3dTshift from AFNI 20160207 (RRID:SCR_005927)[75]. The BOLD time-series (including slice-timing correction when applicable) were resampled onto their original native space by applying transforms to correct for head motion. These resampled BOLD timeseries are referred to as preprocessed BOLD in original space, or just preprocessed BOLD. Then, the BOLD time series were resampled into standard space, generating a preprocessed BOLD run in MNI152NLin2009cAsym space. Finally, we used SPM version 12.0 to smooth the preprocessed BOLD data with 8 mm full-width at half maximum of the Gaussian smoothing kernel.

*Results from the Whole-Brain Offline Analysis of the Localizer Task*

**Table S2.** Brain regions showing greater activity to interference (Type 4) than control (Type 1) trials in the localizer task across all study participants.

| | Peak voxel MNI coordinates | | | T score | P-value | | Cluster size |
|---|---|---|---|---|---|---|---|
| **Region** | **x** | **y** | **z** | **Peak-level** | **Peak-level** | **Cluster-level** | **# Voxels** |
| Occipital Lobe, Lingual Gyrus, Fusiform Gyrus, Inferior Parietal Lobule, Superior Parietal Lobule, Supramarginal Gyrus, Precuneus | 18 | -94 | 2 | 12.21 | 0 | 0 | 4674 |
| | -10 | -96 | 0 | 11.57 | 0 | | |
| | 26 | -78 | -12 | 11.54 | 0 | | |
| Precentral Gyrus | -42 | 0 | 30 | 8.54 | 0 | 0 | 187 |
| | -42 | 26 | 24 | 6.33 | 0.007 | | |
| Middle Frontal Gyrus | -30 | 0 | 54 | 8.29 | 0 | 0 | 126 |
| Dorsal Anterior Cingulate Cortex | 2 | 18 | 48 | 8.25 | 0 | 0 | 144 |

| | | | | | | | |
|---|---|---|---|---|---|---|---|
| | -10 | 14 | 48 | 8.06 | 0 | | |
| Anterior Insula | -34 | 24 | 2 | 7.49 | 0 | 0 | 68 |
| | -36 | 18 | 14 | 6.22 | 0.01 | | |
| Extra-Nuclear | 32 | -24 | -4 | 6.93 | 0.001 | 0 | 13 |
| Inferior Frontal Gyrus | 38 | 6 | 36 | 6.82 | 0.001 | 0 | 67 |
| | 50 | 14 | 32 | 6.12 | 0.013 | | |
| Anterior Insula | 32 | 26 | 0 | 6.78 | 0.002 | 0 | 46 |
| | 32 | 24 | 8 | 6.47 | 0.004 | | |
| Middle Frontal Gyrus | 30 | 2 | 62 | 6.72 | 0.002 | 0 | 51 |
| | 30 | 2 | 54 | 6.61 | 0.003 | | |

*Note.* Clusters are listed in descending order based on cluster size.

**Figure S2.** Brain regions showing greater activity to interference (Type 4) than control (Type 1) trials in the localizer task across all study participants.

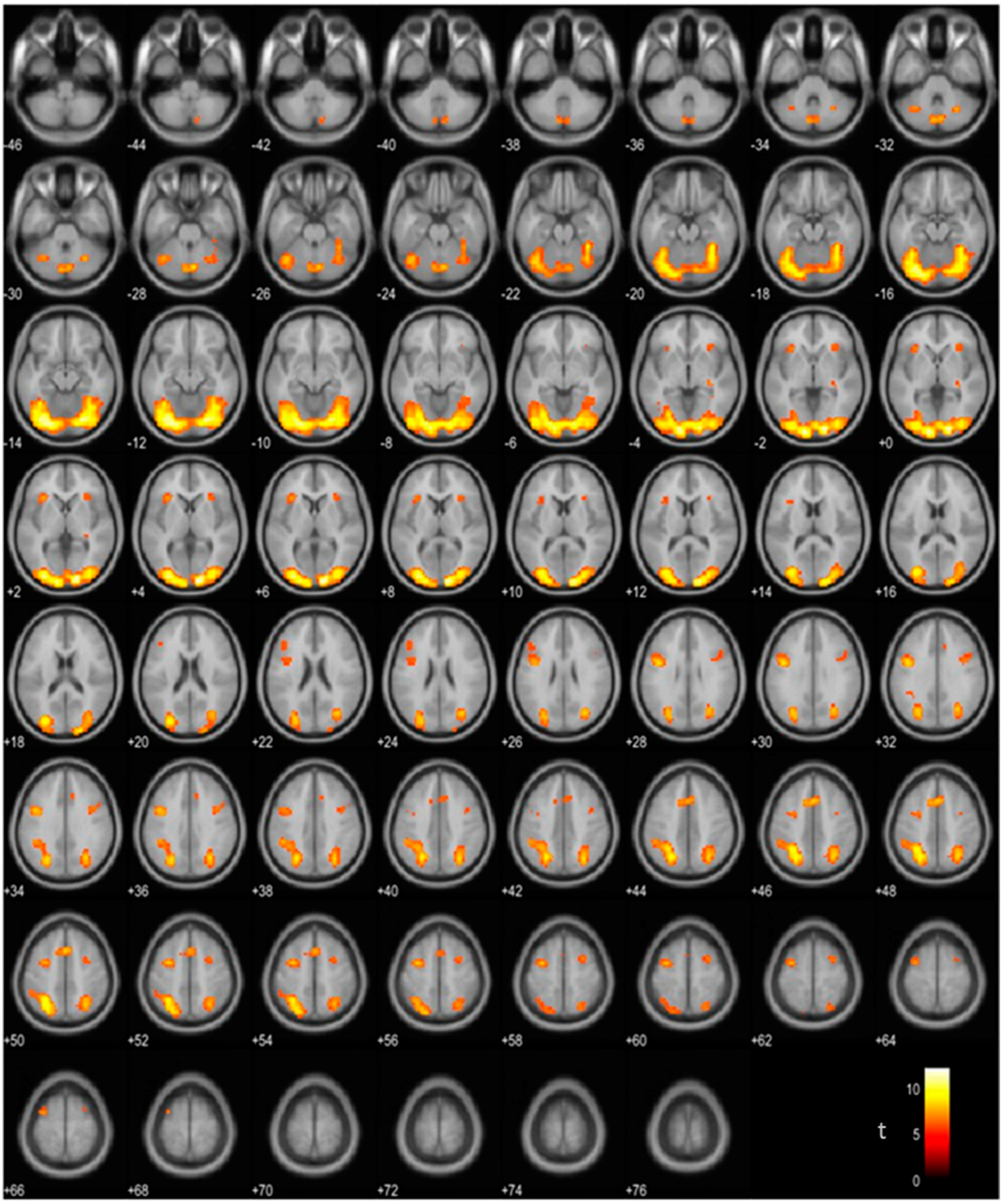


*Note.* Thermometer bar indicates t-scores of voxels above the statistical threshold ($p < 0.05$ FWE corrected).

*Results from the Whole-Brain Offline Analysis of the Neurofeedback Training*

A large cluster consisting of 161,340 voxels was identified with greater activity to stimuli (digits/faces) than fixation during the neurofeedback training runs across all participants.

**Figure S3.** Brain regions showing greater activity to stimuli (digits/faces) than fixation across all runs during the neurofeedback training across all study participants.

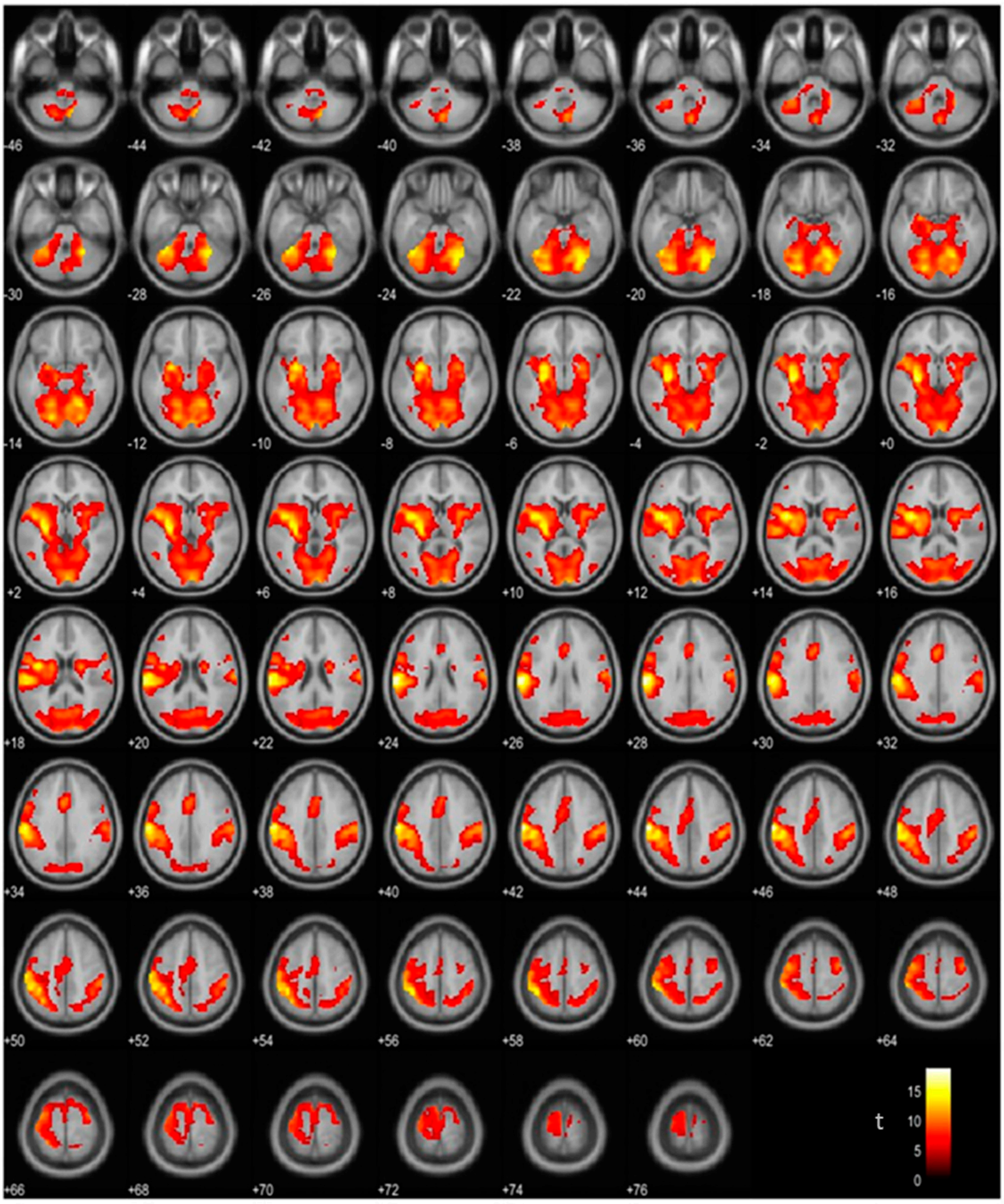

*Note.* Thermometer bar indicates t-scores of voxels above the statistical threshold ($p < 0.05$ FWE corrected).

**Table S3.** Brain regions showing greater activity for older compared to young participants to stimuli (digits/faces) than fixation across all neurofeedback training runs across the two training protocols.

| | Peak voxel MNI coordinates | | | T score | P-value | | Cluster size |
|---|---|---|---|---|---|---|---|
| **Region** | **x** | **y** | **z** | **Peak-level** | **Peak-level** | **Cluster-level** | **# Voxels** |
| Posterior Insula | 45 | -24 | 18 | 6.21 | 0.002 | < 0.001 | 223 |
| | 36 | -18 | 3 | 6.12 | 0.002 | | |
| | 51 | -27 | 30 | 5.96 | 0.004 | | |
| | 39 | 0 | 9 | 5.70 | 0.008 | 0.004 | 15 |
| Middle Frontal Gyrus/ Postcentral Gyrus | 36 | -6 | 63 | 6.39 | 0.001 | < 0.001 | 186 |
| | 48 | -30 | 48 | 6.35 | 0.001 | | |
| | 54 | -18 | 42 | 6.14 | 0.002 | | |
| Cerebellum | 21 | -57 | -24 | 6.51 | 0.001 | < 0.001 | 106 |
| | -3 | -39 | -21 | 6.39 | 0.001 | | |
| Posterior Insula | -33 | -24 | 12 | 6.26 | 0.002 | < 0.001 | 91 |

| | | | | | | | |
|---|---|---|---|---|---|---|---|
| | -33 | -30 | 21 | 6.12 | 0.002 | | |
| | -42 | -39 | 30 | 5.36 | 0.020 | | |
| Postcentral Gyrus | -42 | -27 | 48 | 6.03 | 0.003 | < 0.001 | 41 |
| Fusiform Gyrus | -39 | -48 | -18 | 7.02 | < 0.001 | 0.001 | 27 |
| | -18 | -66 | -51 | 6.08 | 0.003 | 0.001 | 26 |
| Cerebellum Posterior Lobe | -15 | -57 | -54 | 5.72 | 0.007 | | |
| | 3 | -69 | -39 | 5.56 | 0.011 | 0.003 | 17 |
| Supplementary Motor Area | -6 | -15 | 48 | 5.74 | 0.007 | 0.002 | 23 |
| Cerebellum Anterior Lobe | -24 | -54 | -24 | 5.57 | 0.011 | 0.004 | 14 |
| Substantia Nigra, Pars Reticulata | -3 | -15 | -18 | 6.28 | 0.002 | 0.005 | 13 |
| Inferior Frontal Gyrus | 54 | 12 | 33 | 5.81 | 0.006 | 0.007 | 10 |
| | 60 | 9 | 24 | 5.40 | 0.018 | | |

*Note.* Clusters are listed in descending order based on cluster size.

**Figure S4.** Brain regions showing greater activity for older compared to young participants to stimuli (digits/faces) than fixation across all neurofeedback training runs across the two training protocols.

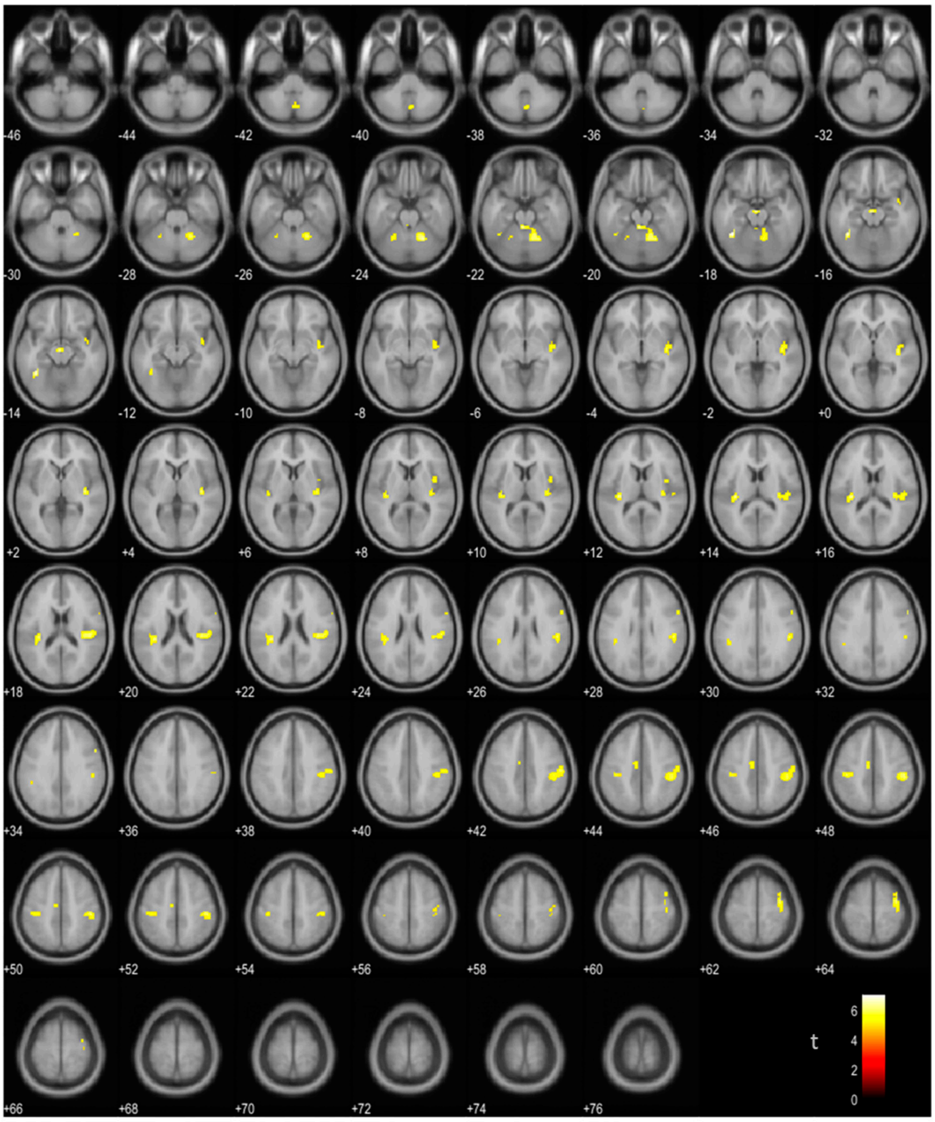


*Note.* Thermometer bar indicates t-scores of voxels above the statistical threshold ($p < 0.05$ FWE corrected).

**Table S4.** Parameter estimates ($B/\sigma^2$ (SE), Standard Error [SE]) of effects for reward points, dACC BOLD signal, reaction time, and response accuracy.

| | **Reward Points** | **dACC BOLD Signal** | **Reaction Time** | **Response Accuracy** |
|---|---|---|---|---|
| *Fixed Effect* | *B* (*SE*) | *B* (*SE*) | *B* (*SE*) | *B* (*SE*) |
| Experimental vs. Age Control | **16.91 (7.94)** | -0.0042 (0.026) | **-0.246 (0.059)** | **5.49 (2.65)** |
| Experimental vs. Inverse Condition Control | **-17.47 (8.22)** | -0.0197 (0.0269) | -0.092 (0.061) | -1.78 (2.74) |
| Experimental vs. Young Down-Regulation | -4.18 (8.22) | -0.0115 (0.0269) | **-0.172 (0.061)** | 3.65 (2.74) |
| Session | **4.14 (1.13)** | **0.0012 (0.0005)** | **-0.008 (0.003)** | **0.62 (0.13)** |
| Session: Experimental vs. Age Control | **-5.83 (1.89)** | **-0.0021 (0.0009)** | **0.009 (0.004)** | **-0.55 (0.22)** |
| Session: Experimental vs. Inverse Condition Control | **-7.17 (1.96)** | **-0.003 (0.0009)** | -0.009 (0.005) | **-1.15 (0.23)** |
| Session: Experimental vs. Young Down-Regulation | **-4.77 (1.96)** | -0.0003 (0.0009) | 0.0001 (0.005) | 0.42 (0.23) |
| Run | -0.7 (1.26) | 0.0001 (0.0006) | 0.002 (0.003) | **0.29 (0.15)** |
| Run: Experimental vs. Age Control | **-9.51 (2.11)** | 0.0011 (0.001) | 0.003 (0.005) | -0.46 (0.25) |
| Run: Experimental vs. Inverse Condition Control | 2.19 (2.19) | -0.0019 (0.001) | 0.001 (0.005) | -0.43 (0.25) |

| | | | | |
|---|---|---|---|---|
| Run: Experimental vs. Young Down-Regulation | 2.79 (2.19) | 0.0011 (0.001) | -0.0001 (0.005) | -0.27 (0.25) |
| Session × Run | 0.28 (0.83) | -0.0003 (0.0004) | -0.002 (0.002) | **-0.21 (0.10)** |
| Session × Run: Experimental vs. Age Control | 1.48 (1.39) | 0.0001 (0.0007) | 0.004 (0.003) | 0.21 (0.16) |
| Session × Run: Experimental vs. Inverse Condition Control | 1.8 (1.44) | 0.0006 (0.0007) | 0.003 (0.003) | **0.42 (0.17)** |
| Session × Run: Experimental vs. Young Down-Regulation | -1.57 (1.44) | 0.0003 (0.0007) | 0.00005 (0.003) | 0.13 (0.17) |
| Intercept | **53.82 (4.74)** | **1.047 (0.0158)** | 1.034 (0.035) | **29.11 (1.58)** |
| *Random Effect* | *$\sigma^2$ (SE)* | *$\sigma^2$ (SE)* | *$\sigma^2$ (SE)* | *$\sigma^2$ (SE)* |
| Intercept | **332.28 (84.54)** | **0.0042 (0.0009)** | **0.022 (0.005)** | **44.08 (9.4)** |

Note. We examined effects of session and run, as well as their interaction, on reward points, dACC BOLD signal, reaction time, and response accuracy via four separate models, with training group as moderator. The experimental group served as reference category, and therefore effects of session and run, as well as their interaction, reflect those in the experimental group. Bold print indicates significant effects at $p < 0.05$.

*Visualization of Run Effects Within Neurofeedback Training Sessions for All Outcomes Variables and in all Groups*

**Figure S5.** Marginal estimates for Reward Points (*Panel A*), dACC BOLD Signal (*Panel B*), Reaction Time (*Panel C*), and Response Accuracy (*Panel D*) over the course of neurofeedback training *runs* by *sessions* for the experimental group (black circles), the age control group (gray circles), the inverse condition control group (black diamonds), and the young down-regulation group (gray diamonds). Alternating pattern of gray and white background shades reflect the seven training sessions across the training protocol. Theoretical range of the y-axis was 0 to 360 for Reward Points (*Panel A*) and 0 to 36 for Response Accuracy (*Panel D*); note that due to a ceiling effect, error bars for all sessions in the age control group and young down-regulation group exceeded the theoretical maximum for Response Accuracy (*Panel D*). Error bars indicate 95% confidence intervals.

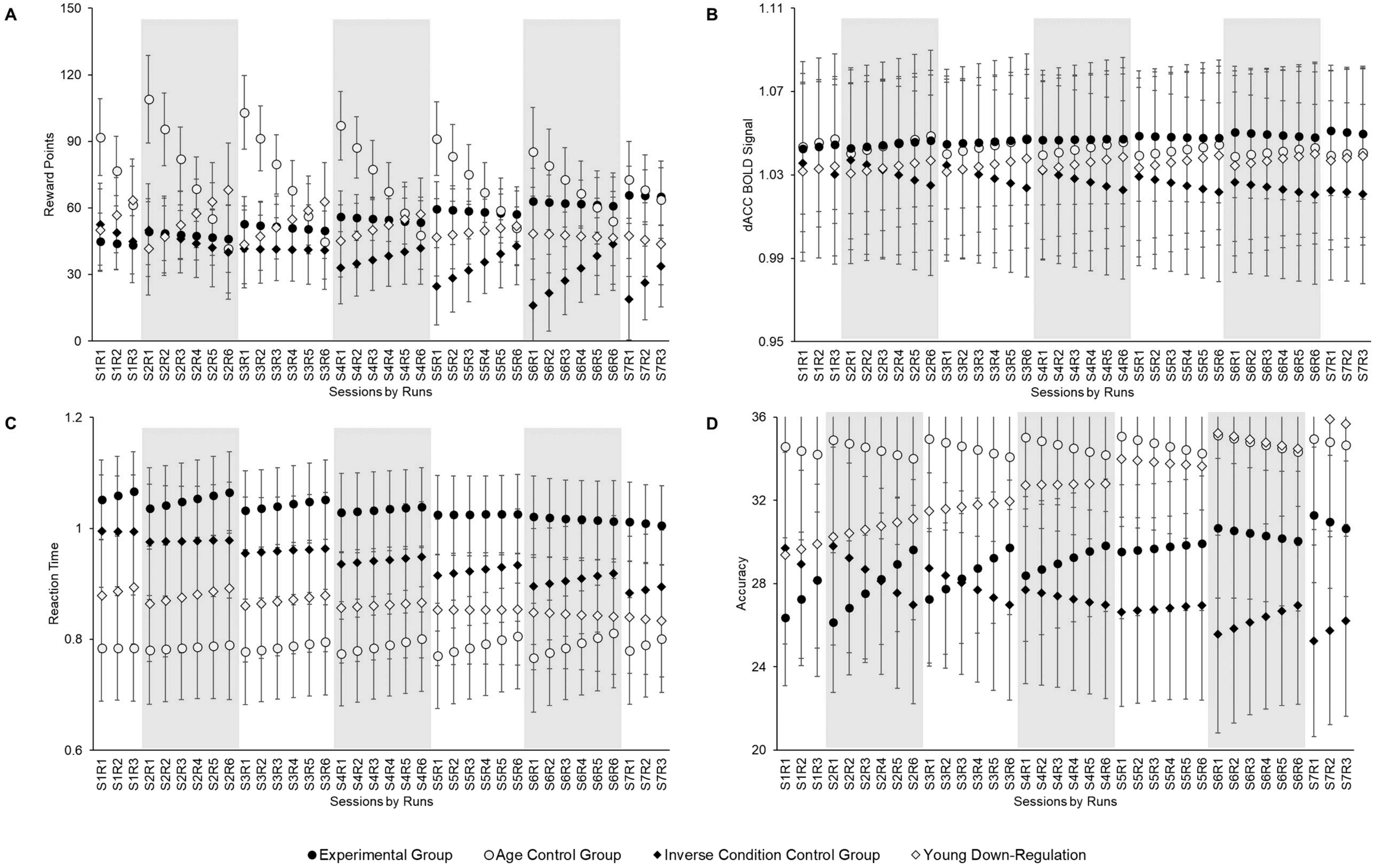


As reported in the manuscript, for reward points the effect of run was significant in the age control group ($B$ = -10.21, $z$ = -5.52, $p < 0.001$) and for response accuracy the effect of run ($B$ = 0.29, $z$ = 1.96, $p$ = 0.05) and its interaction with session ($B$ = -0.21, $z$

= -2.12, $p = 0.03$) were significant in the experimental group. No other effects of, or interactions with, run were significant (all *p*s > 0.05).

*Analysis of Self-Reported Motivation Comparing the Experimental Group with the Inverse Condition Control and the Young Down-Regulation Groups*

Findings from a multilevel regression model on self-reported motivation comparing the experimental group vs. the inverse condition control as well as the young down-regulation group were as follows: The effect of session in the experimental group was significantly different from the session effect in the inverse condition control group ($B$ = -1.21, $z$ = -2.74, $p$ = 0.006) but not the young down-regulation group ($B$ = 0.55, $z$ = 1.24, $p$ = 0.22). Furthermore, the effect of run in the experimental group was significantly different from the run effect in both the inverse condition control group ($B$ = -1.31, $z$ = -2.66, $p$ = 0.008) and the young down-regulation group ($B$ = -2.98, $z$ = -6.08, $p < 0.001$).

Follow-up analysis within the inverse condition control group showed that self-reported motivation in this group did not only decrease across the runs within a given session ($B$ = -1.81, $z$ = -5.34, $p < 0.001$) but also decreased from session to session ($B$ = -1.20, $z$ = -3.99, $p < 0.001$). Follow-up analysis within the young down-regulation group showed a linear decrease in self-reported motivation across runs ($B$ = -3.48, $z$ = -7.79, $p < 0.001$).
